# Seeing Ghosts: A Quality Improvement Intervention to Decrease Phantom Scanning Through Increased Image Archiving of POCUS by Internal Medicine Residents

**DOI:** 10.24908/pocusj.v10i01.17775

**Published:** 2025-04-15

**Authors:** Linden Kolbenson, Talha Salman, Amanda Oro, Paul Olszynski

**Affiliations:** 1 Department of Medicine, University of Saskatchewan, Saskatoon, SK, CAN; 2 Department of Emergency Medicine, University of Saskatchewan, Saskatoon, SK, CAN

**Keywords:** quality improvement, point of care ultrasound, image archiving, internal medicine, medical education

## Abstract

Point of care ultrasound (POCUS) is used in internal medicine (IM) to augment clinical decision making and improve procedural safety. Institutionally-supported archiving software can help learners track scan numbers and receive feedback on image acquisition and interpretation. At the University of Saskatchewan, IM residents use POCUS for procedures and assessments but rarely save images, limiting feedback opportunities. Our quality improvement project aimed to increase the number of POCUS images saved by Postgraduate Year One (PGY-1) IM residents, targeting over 75% of non-procedural scans and ensuring over 50% of residents save at least one scan. This quality improvement project was conducted on a clinical teaching unit at an academic hospital over two years. We used four Plan-Do-Study-Act (PDSA) cycles each year to measure the percentage of non-procedural scans saved by PGY-1 IM residents. As a balance measure, we compared the number of scans performed historically and during the study period to monitor for changes in usage. Data was collected using an ultrasound sign-out sheet. At baseline, no diagnostic scans were saved by PGY-1 IM residents. Post-intervention, 56% of scans were archived in cohort one and 76% in cohort two. Additionally, 79% of residents in cohort one and 94% in cohort two archived at least one scan. The balance measure improved from 1.13 in the first year to 2.25 in the second, suggesting image archiving is not a deterrent to performing scans. Through this intervention, we significantly increased the archiving of non-procedural scans by PGY-1 IM residents. We advocate for implementing a formal POCUS archiving system to promote quality assurance in residency programs.

## Background

Point of care ultrasound (POCUS) use is becoming ubiquitous within the practice of internal medicine (IM) and its subspecialties. It has improved diagnostic and procedural accuracy, which makes it an attractive tool to inform clinical decision making and guide care [1]. Given the well-documented utility of POCUS, structured training is now being integrated into Canadian IM residency programs [2–4]. Literature supports the need for formalized POCUS education in IM residency. Alongside didactic teaching and supervised scanning, image archiving features prominently [5]. This is consistent with recommendations from other specialty societies [6–13]. Imaging archiving software allows learners to track scan numbers and receive feedback on image acquisition and interpretation for scans completed independently. Programs can further use image archiving for quality assurance purposes.

Multiple barriers exist to implementation and use of a POCUS image archiving system. One of the biggest barriers is the high cost of POCUS archiving software. This can exceed CAD$25,000 per year, not including the annual cost of local server space for image storage, where applicable [14]. Other barriers include time restraints and lack of training. Physicians may have limited time to properly archive images during their clinical work, insufficient training on how to use image archiving systems effectively, and lack familiarity or support for such systems within a hospital's information technology ecosystem [15]. A necessary step to utilizing image archiving software is to have learners save scans when they perform POCUS. “Phantom Scanning” is a known and problematic phenomenon in POCUS where clinicians scan the patient without saving representative images [16,17]. Without a culture and workflow conducive to image capture, benefits of image archiving are lost. Currently, there is a paucity of literature related to improving the number of POCUS scans saved by both learners and practicing physicians. Prior work focuses on improving documentation and POCUS workflow in general among emergency physicians [18–21]. Discovering additional strategies that improve the number of scans saved by learners has the potential to improve POCUS use locally and at other programs building their POCUS training program. With this in mind, our team sought to improve POCUS use at our centre by focusing on interventions that increase the number of scans saved by residents.

IM residents at the University of Saskatchewan use POCUS for procedures and clinical assessment of patients. To begin our quality improvement project, we first defined what current POCUS use looks like among our residents. Retrospective data collection from the 2021-2022 academic year showed that among Postgraduate Year One (PGY-1) IM residents, POCUS was used 94 times. Non-procedural indications accounted for 32% of use, while procedural indications accounted for 24% of use. No indication was documented in 44% of cases. In all 94 uses, no images were saved. The objective of this quality improvement project was to show improvement in the number of scans saved by PGY-1 IM residents. Our specific project aim was to have >75% of all non-procedural POCUS scans saved and have >50% of IM PGY-1 residents saving non-procedural POCUS images.

## Methods

### Study Setting and Design

This quality improvement project was conducted in a single academic hospital on the IM clinical teaching unit (CTU). Two sequential cohorts of PGY-1 IM residents were involved, including 19 residents in the 2022/2023 academic year and 17 residents in the 2023/2024 academic year. Residents completed six blocks of CTU throughout the academic year split between three teams: two ward teams and an emergency department team. Since 2019, each PGY-1 resident has received dedicated POCUS training totaling nine hours of procedural guidance practice (POCUS-guided venous access) and twenty-hours of education in core POCUS applications (ten-hours of supervised scanning). Between July 2022 and June 2023, four Plan-Do-Study-Act (PDSA) cycles were implemented based on the Institute for Healthcare Improvement (IHI) Model for Improvement [22] for cohort one. Between July 2023 and June 2024, four PDSA cycles were implemented for cohort two. PGY-1 residents were chosen because local data indicated they were the most frequent users of POCUS on the IM CTU, which would assure adequate amounts of data for analysis. Residents beyond PGY-1 have limited use of POCUS on CTU owing to their supervisory role and more time spent away on subspecialty and elective rotations. Ultrasound use on CTU was chosen for practical reasons as that is where our ultrasound machines were located. The report of this work is outlined here, according to the Standards for Quality Improvement Reporting Excellence 2.0 guidelines [23].

### Measures and Aims

Retrospective data analysis revealed that the baseline number of saved scans was 0%. Our project aim was to have >75% of all non-procedural POCUS scans performed by PGY-1 IM residents saved by the end of the quality improvement project study period. Our primary outcome measure was the percentage of non-procedural scans saved per total number of non-procedural scans performed by PGY-1 IM residents. A secondary outcome measure was the percentage of PGY-1 IM residents who had saved at least one non-procedural scan during the study period. Our balancing measure was searching for any decrease in the use of POCUS due to a new expectation to save scans. This was achieved by calculating a ratio of non-procedural scans performed in the project period to non-procedural scans performed in the prior academic year. Measures were evaluated every four weeks over a 100-week period.

### Data Collection

**Figure 1.**
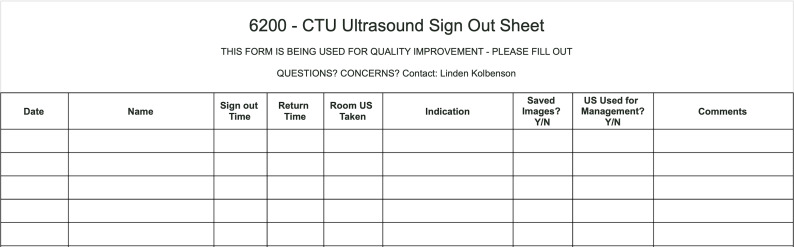
Ultrasound sign-out sheet used for data collection for non-emergency department based clinical teaching unit (CTU) teams.

**Figure 2.**
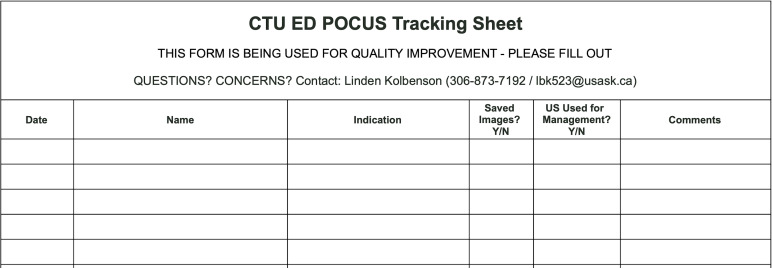
Ultrasound tracking sheet used for data collection for the emergency department based clinical teaching unit (CTU) team.

An ultrasound sign-out sheet was created and used to collect relevant data (Figure 1). During the 2022-2023 academic year, the sign-out sheet was posted at the ultrasound machine for two of the CTU teams. At the start of the 2023-2024 academic year, an additional tracking sheet was posted in the emergency department for the final CTU team (Figure 2). Data from the sign-out and tracking sheets was collected each block and entered in an online spreadsheet for analysis. Data from the sign-out and tracking sheets regarding saved scans were checked against the internal memory of the ultrasound machines.

### Intervention Design and Implementation

**Figure 3.**
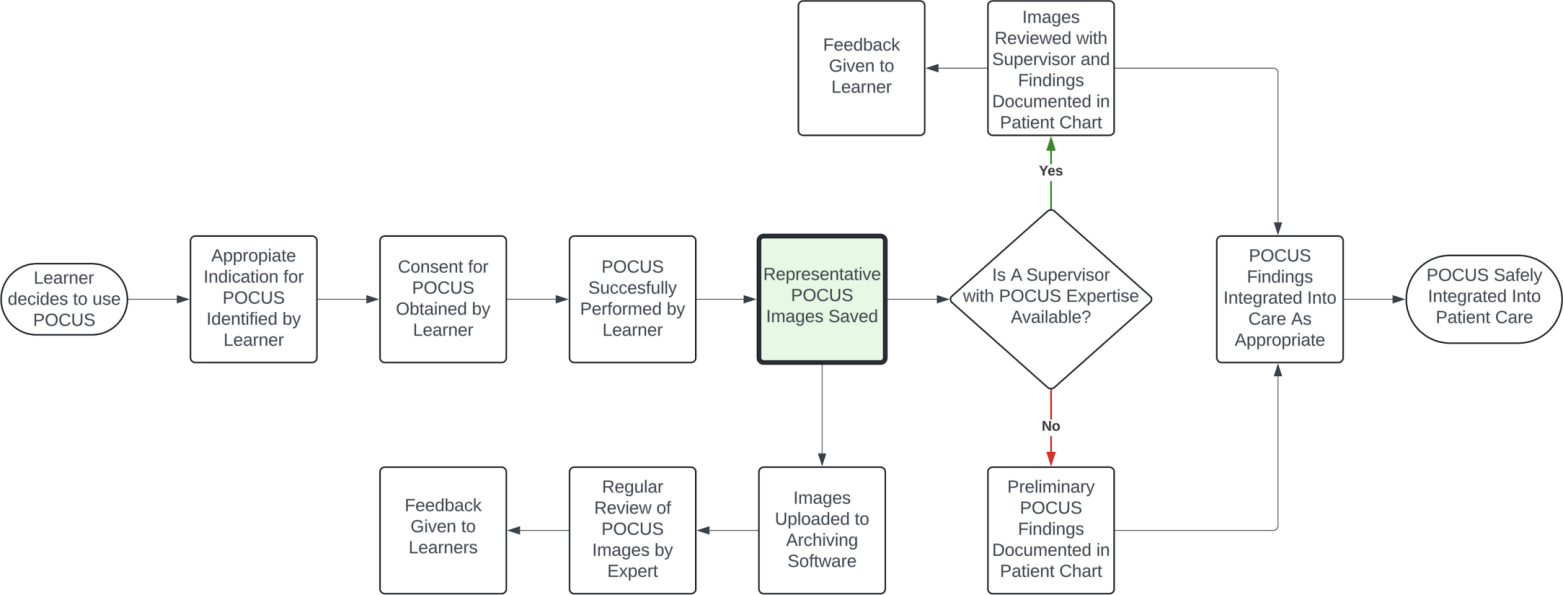
Process map of proposed ideal point of care ultrasound (POCUS) use on a clinical teaching unit (CTU) for internal medicine (IM) residents.

**Figure 4.**
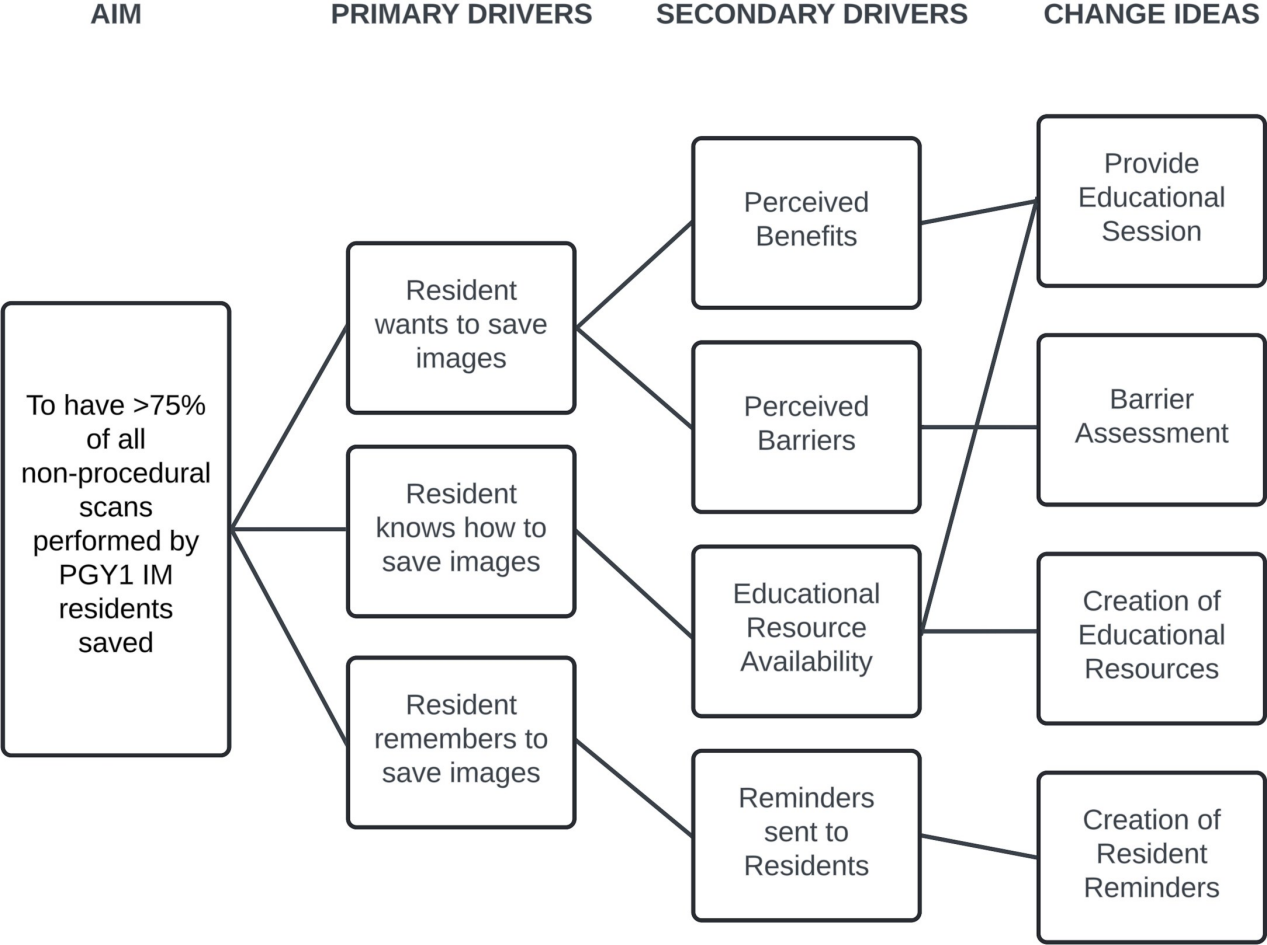
Key driver diagram.

Prior to intervention design, a process map of proposed ideal POCUS use by IM residents was developed to identify areas of opportunity for quality improvement (Figure 3). An intervention that targeted the process of saving representative POCUS images was chosen. Next, a key driver diagram was created to identify change ideas (Figure 4). Based on our key driver diagram, a series of PDSA cycles were implemented over the study period with a focus on resident education.

## Cohort 1 (Academic year 2022-2023)

### Plan-Do-Study-Act 1

Our first intervention was an in-person education session with incoming PGY-1 IM residents prior to the start of the academic year. With support from the IM residency program director, this session was integrated into a pre-existing “resident bootcamp” week. The overall objective of the session was to orient residents to the ultrasound machines and the sign-out sheet. This included an in-person tutorial on how to save scans. Motivations for saving scans and the benefits of image archiving were discussed. Informal feedback was elicited during the session regarding potential barriers residents might face to saving scans. There was a common desire for written and visual resources that outlined the process of saving scans on the different ultrasound machines. Attendance was used as a fidelity measure and was 100%.

### Plan-Do-Study-Act 2

The second cycle was the creation of a written educational resource that demonstrated the process for archiving scans locally on our ultrasound machines. This resource was distributed via our training program resident website.

### Plan-Do-Study-Act 3

The third cycle was the creation of video resources that demonstrated the process for archiving scans on our ultrasound machines. This resource was distributed via email and through our training program resident website.

### Plan-Do-Study-Act act 4

The fourth cycle was soliciting feedback from residents with the lowest percentage of saved scans for potential barriers they were experiencing.

## Cohort 2 (2023-2024 Academic Year)

### Plan-Do-Study-Act 5

Similar to PDSA 1, an in-person education session was held for incoming PGY-1 residents. In addition to content discussed in PDSA 1, residents were oriented to additional data collection sign-out sheets and resources created during PDSA cycles 1-4. Attendance was used as a fidelity measure and was 100%.

### Plan-Do-Study-Act 6

Additional video and written resources were created and distributed to residents, including new signage to capture data from the CTU in the emergency department. QR codes were placed on each ultrasound machine that linked to resources on how to save images. These also served as a visual reminder to save scans. Views on the video content created served as a fidelity measure.

### Plan-Do-Study-Act 7

Text messages were sent to residents with a summary of their POCUS use including total number of scans performed and percentage of scans saved. Feedback was sought from residents regarding ongoing barriers to saving scans. Resident responses to the text messages were used as a fidelity measure.

### Plan-Do-Study-Act 8

Reminder emails were sent to residents beginning a new block on the CTU. The reminder email included links to resources on how to save scans.

## Analysis

**Figure 5.**
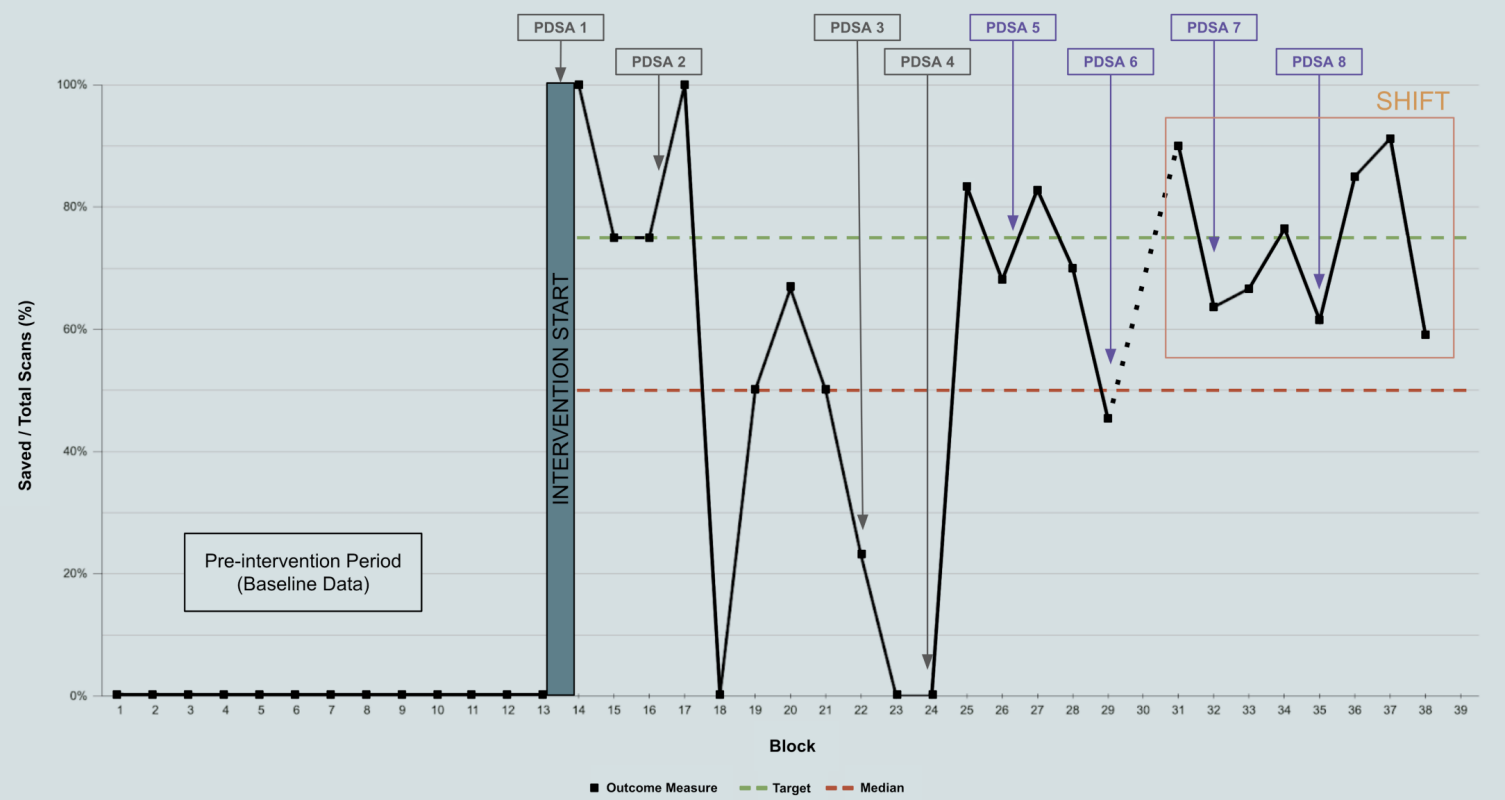
Run chart for the study period. Approximate implementation times of change ideas and Plan-Do-Study-Act (PDSA) cycles are shown. A shift in a positive direction is indicated by the orange box. The dotted line indicates a period with no ultrasound use.

A run chart was used to track our primary outcome measure over time (Figure 5). Special cause variation was defined following the IHI rules. The primary outcome measure was depicted as baseline vs post-intervention percentage. Baseline data came from PGY-1 IM resident data from the academic year prior to the intervention.

## Results

A summary of data is presented in Table 1. Baseline data indicated 0% of non-procedural scans were saved and no PGY-1 IM residents had saved a single scan. Post intervention, a total of 80 non-procedural POCUS scans were performed by PGY-1 IM residents in cohort one and 180 in cohort two. The amount of non-procedural scans archived was 56% and 76% in cohort one and two, respectively. At least one scan was archived by 79% of residents in cohort one and 94% in cohort two. Our final balance measure was 1.13 for the first year and 2.25 for the second, indicating an increase in scans performed during the study period as compared to the prior academic year. Our intervention met criteria for special cause variation with a shift in a positive direction as showcased in our run chart (Figure 5).

**Table 1.** Summary of data.

	Non-Procedural Scans Saved	Non-Procedural Scans Performed (Number)	Outcome Measure (%)	Residents Saving ≥ 1 Scan (%)	Balance Measure
**Baseline**	0	30	0	0	N/A
**Cohort 1**	45	80	56	79	1.13
**Cohort 2**	136	180	76	94	2.25
**Total**	181	260	70	87	N/A

## Discussion

To establish best practices within our local IM POCUS curriculum, we performed a quality improvement project spanning two years. The goal was to increase the number of POCUS scans archived by PGY-1 IM residents. We observed an increase in our primary and secondary outcome measures, raising the number of saved scans from 0% to 70% over the study period with no decrease in the number of scans performed. The largest impact of our intervention came from a combination of addressing knowledge gaps regarding image archiving and reminding residents to save their scans. Success was achieved through an iterative quality improvement process. Statistically significant changes were seen in the second cohort with a positive shift related to refinement of our intervention over multiple PDSA cycles.

Since the number of saved scans pre-intervention was 0%, our post-intervention increase may appear extreme. The lack of saved scans pre-intervention can be explained by a few factors. Historically, residents did not know how or why to save images. Until this project, educational sessions within the POCUS curriculum did not emphasize the process or importance of image archiving. The majority of scans performed by residents were done for educational purposes under the indirect supervision of a staff with limited POCUS training. Finally, due to the lack of formal image archiving software, residents could only receive feedback on saved scans through a time-consuming process of downloading images and then uploading to cloud-based image archiving solutions. All these factors led to a culture where image archiving, in the backdrop of a busy clinical service, was not deemed sufficiently important or worthwhile.

The biggest lesson learned from this project was that residents require reminders. This is not a surprise given all the competing interests inherent in completing the first year of IM residency. Although not meeting special cause variation, it would appear that our process measure would increase immediately post-implementation of the PDSA cycle with it decreasing over time until the next cycle. This would suggest that continuously reminding residents to save images is required for consistent results. This finding also highlights the need to have POCUS leaders within programs who can ensure residents are reminded and kept on track with image archiving.

The major strength of this quality improvement project is the use of straightforward interventions. Other IM programs could easily implement similar knowledge and reminder strategies to improve local POCUS use.

There are multiple limitations to our study. Though our PDSA interventions focused on improving literacy around image archiving and giving residents continual reminders, we acknowledge that there may be other contributing factors influencing our results. A resident's confidence and familiarity with POCUS could make them more inclined to save scans irrespective of our intervention. Some residents may start PGY-1 with more POCUS training depending on where they completed medical school. These residents may skew the data in our favour.

However, there was an absence of a clear trend in increased image archiving as the academic year progressed as one would expect more familiarity near the end of the academic year. Additionally, residents may be using POCUS to inform their clinical decisions despite inadequate training and supervision. A resident may be more inclined to save their image if it is being used to inform management decisions. As well, our study only included POCUS use within the PGY-1 IM cohort. This could introduce selection bias and limit generalizability to learners beyond PGY-1 and IM programs. Further, our intervention required routine check-ins with residents, making this protocol labourious. Implementation may be challenging in residency programs with significantly larger volumes of trainees; however, automation could address this.

Data collection also introduced several limitations. The use of a sign-out sheet limited the accuracy and reliability of our data. We assumed that the resident who signed out the machine was the one that used it, that all use was captured with the sign-out sheet, and that information was recorded accurately. In reality, a resident could have signed out the machine for a colleague or forgot to sign it out altogether. Additionally, the nature of their scan could have been incorrectly reported on the sign out sheet. We did not choose to include which specific types of POCUS scans were being performed, potentially missing important information on which scan types were more amenable to archiving. Additionally, data collection was not the same throughout the study period with the introduction of data from the CTU emergency department team in the second cohort. A lack of fidelity measures was also a limitation of the study. It was not possible to track how many residents received and read reminder emails, whether views on educational videos were from PGY-1 IM residents, and if written educational materials were used by residents.

## Conclusion

Our team improved PGY-1 IM resident POCUS use in regard to saving POCUS images. Both the number of scans saved and the number of residents saving scans increased, without decreasing the number of scans performed by residents. We are hopeful that the results of this intervention will inform future policy in our IM program. We will advocate for education about image archiving in our current IM POCUS curriculum. Our quality improvement process will continue with the next cohort of incoming PGY-1 IM residents. We will specifically test how automating data collection and intervention implementation effects image archiving and will attempt to include a more diverse group of residents across more training levels and subspecialties. With these future research directions, we hope to improve implementation and generalizability of our intervention.

At our local institution we currently do not have any departmental image archiving software. However, we continue to advocate for this technology and are nurturing an image-archiving culture in preparation for this eventuality. By documenting a clear increase in the number of archived scans through this quality improvement project, we hope to advocate for implementation of a formal POCUS image archiving system. With such a system in place, physicians and residents can be encouraged to optimize image acquisition and to carefully consider what is being demonstrated through a scan [14]. This self-awareness encourages active feedback and review from POCUS experts, thereby furthering quality assurance around POCUS programs.
